# Determining Frequent Patterns of Copy Number Alterations in Cancer

**DOI:** 10.1371/journal.pone.0012028

**Published:** 2010-08-12

**Authors:** Franck Rapaport, Christina Leslie

**Affiliations:** Computational Biology Program, Sloan-Kettering Institute, New York, New York, United States of America; Virginia Tech, United States of America

## Abstract

Cancer progression is often driven by an accumulation of genetic changes but also accompanied by increasing genomic instability. These processes lead to a complicated landscape of copy number alterations (CNAs) within individual tumors and great diversity across tumor samples. High resolution array-based comparative genomic hybridization (aCGH) is being used to profile CNAs of ever larger tumor collections, and better computational methods for processing these data sets and identifying potential driver CNAs are needed. Typical studies of aCGH data sets take a pipeline approach, starting with segmentation of profiles, calls of gains and losses, and finally determination of frequent CNAs across samples. A drawback of pipelines is that choices at each step may produce different results, and biases are propagated forward. We present a mathematically robust new method that exploits probe-level correlations in aCGH data to discover subsets of samples that display common CNAs. Our algorithm is related to recent work on maximum-margin clustering. It does not require pre-segmentation of the data and also provides grouping of recurrent CNAs into clusters. We tested our approach on a large cohort of glioblastoma aCGH samples from The Cancer Genome Atlas and recovered almost all CNAs reported in the initial study. We also found additional significant CNAs missed by the original analysis but supported by earlier studies, and we identified significant correlations between CNAs.

## Introduction

Cancers are a complex set of proliferative diseases whose progression, in most cases, is driven in part by an accumulation of genetic changes, including copy number aberrations (CNAs) of large or small genomic regions [1], [2], [3] which may for example lead to amplification of oncogenes or loss of tumor suppressor genes. However, cancer progression is also often characterized by increasing genomic instability, potentially generating many “passenger” CNAs that do not confer clonal growth advantage. These processes give rise to a complicated landscape of genomic alterations within an individual tumor and great diversity of these CNAs across tumor samples, making it difficult to identify driver mutations associated with cancer progression.

In recent years, array-based comparative genomic hybridization (aCGH) [4], [5] and single nucleotide polymorphism (SNP) arrays [6] have been used to analyze the CNAs of tumor samples at a genomic scale and at progressively higher resolutions. Moreover, numerous large-scale tumor profiling studies have generated copy number data sets for large cohorts of tumors [7], [8]. These large and complex “cancer genome” data sets present difficult statistical challenges [9]. Individual CNAs may be as small as a few adjacent probes or as large as a whole chromosomes and may be difficult to detect above probe-level noise; moreover, it is unclear how to make sense out of diverse CNAs from hundreds of tumors.

Typically, two kinds of analyses have been carried out on copy number data sets:

1. clustering of samples by their CNAs, to determine possible tumor subtypes characterized by a common pattern of amplifications and deletions;
2. determining significant genetic aberrations, either gains or losses, that occur frequently in the data set, since these may represent driver mutations important for tumor progression.

Almost always, these problems are tackled with a pipeline approach, where aCGH profiles of chromosomes for individual samples are first processed by a segmentation algorithm; individual segments (genomic regions) are “called” as gains or losses, based on their amplitude, using a choice of statistical procedure and significance threshold; and finally the called segments are used as input to a clustering algorithm [1], [10], [11] or score-based method for determining significant common aberrations [12], [13], [14]. The disadvantage of pipeline approaches, however, is that algorithmic choices and tuning parameters at each step may produce very different results, and mistakes or biases are propagated forward.

For the first step, there are numerous segmentation algorithms [15], [16], [17], [18] that yield significantly different segment boundaries [19], leading to different calls of gains and losses. The final step of analyzing CNAs across samples depends critically on choices made earlier. As an example, the widely-used GISTIC method for determining frequent aberrations [12] uses as its test statistic, at each locus, the number of samples in which a gain (or loss) is present multiplied by the mean amplitude of the gain (loss). However, both the count and the mean amplitude depend on earlier choices in the pipeline.

In this study, we propose a novel and mathematically robust method for finding significant patterns of CNAs in a large copy number data set directly from the probe-level data. By avoiding a pipeline approach involving a segmentation step, our algorithm exploits probe-level correlations in aCGH data to discover subsets of samples that display common CNAs. By applying the approach in a hierarchical fashion to iteratively partition the data set, we discover both large- and small-scale events and can detect statistically significant CNAs occurring on 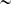
5% of the samples. In this way, the algorithm addresses both the clustering problem and the frequent aberration problem at the same time. Algorithmically, our approach is related to recent work on maximum-margin clustering [20], [21], [22], [23], which extends support vector machine-like optimization approaches to the problem of unsupervised clustering. That is, each partition of the data set is achieved by learning a linear classifier of the probe-level aCGH profiles that assigns samples to one group or the other. We also build on ideas developed for supervised classification of aCGH samples [24], [25], [26], [27], in particular, the use of piece-wise constant and lasso [17], [26], [28] regularization terms in the optimization problem, which encourages the classifier to make decisions using only a small number of probes in informative contiguous regions.

We tested our approach on a large cohort of glioblastoma aCGH samples recently generated by The Cancer Genome Atlas Project (TCGA) [7]. We found that the major CNAs detected by our algorithm are largely consistent with the original TCGA study, in that almost all CNAs previously reported were also in our results. However, we found additional significant CNAs missed by the TCGA analysis but supported by earlier studies and/or expression analyses. Moreover, the hierarchical partitioning approach summarizes the set relationships and dependencies between different CNAs, which may be helpful for generating hypotheses about the sequence of CNAs in tumor progression.

## Results

### Algorithm overview

**Figure 1:**
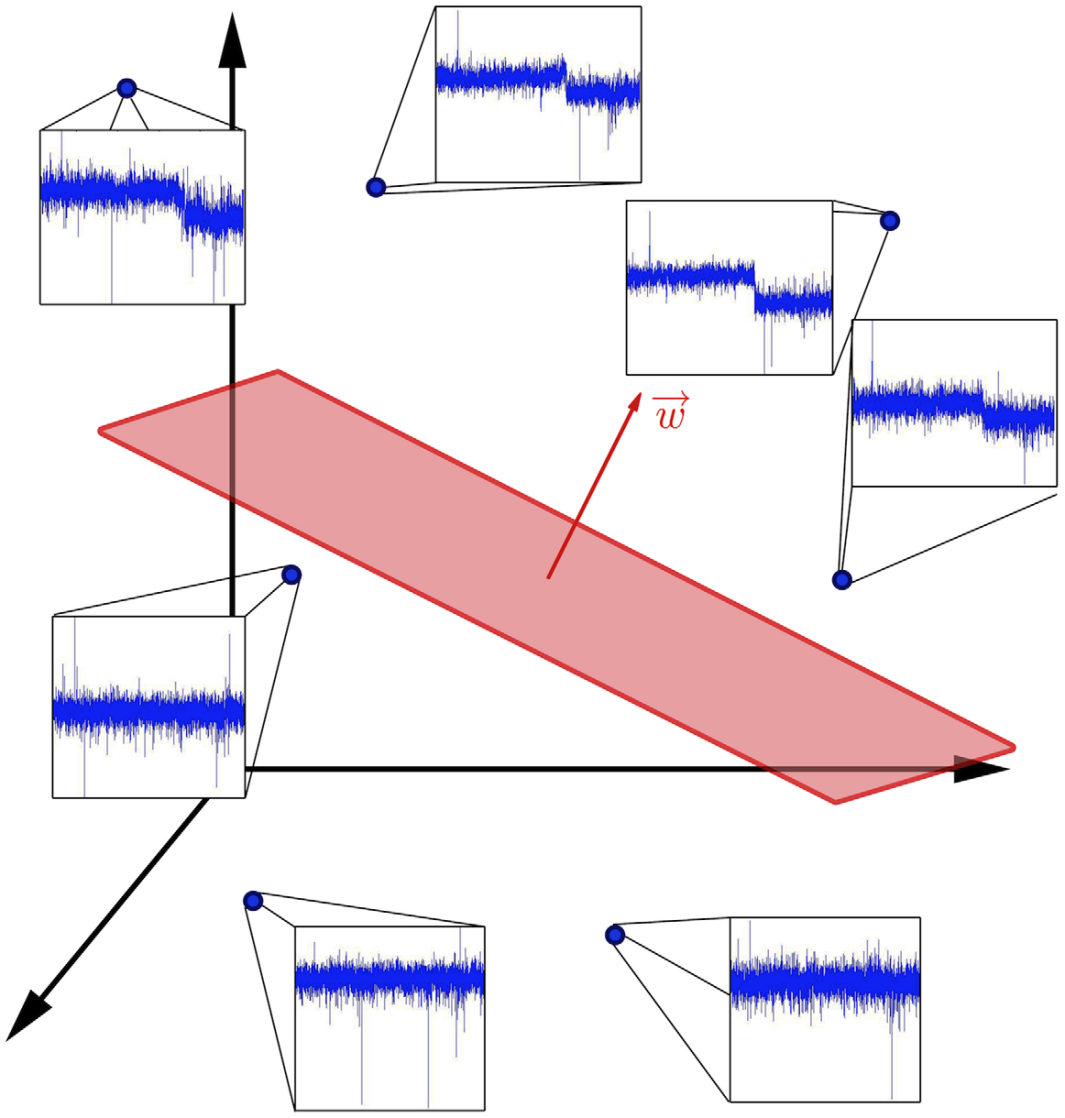
Toy representation of a linear partition of aCGH samples using large-margin techniques. The algorithm finds a linear function 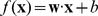 that is able to partition the aCGH samples into two groups. By solving an optimization problem, the algorithm determines the vector 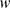, which geometrically represents the normal vector of a hyperplane (shown in red) separating the samples, along with the bias term 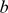, and the assignment of samples to groups. In the toy example shown, the hyperplane separates the samples that present a deletion on the q arm (above the hyperplane) from the ones that do not (below the hyperplane).

Our algorithm iteratively partitions a data set of tumor aCGH profiles for a given chromosome to discover subsets of tumors with similar CNAs. Instead of using standard preprocessing techniques like segmentation algorithms, we directly use probe-level data and incorporate prior knowledge about the nature of this data, namely: (1) successive probes are correlated, i.e. are likely to represent the same copy numbers; and (2) a chromosome typically (though not always) harbors few CNAs. At each partitioning step, we learn a linear separator 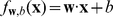 that assigns aCGH profiles 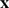 to one of two classes, represented geometrically by the two half-spaces (i.e. 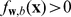 and 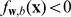) on either side of the hyperplane defined by the normal vector 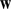 and bias term 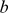 (Figure 1). Here, chromosome profiles 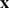 and the weight vector 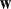 are real-valued vectors with dimension equal to the number of probes for the chromosome, and 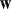 is determined by solving an optimization problem (see Methods) where it is constrained to be piecewise constant (successive probes tend to have the same weights) and sparse (few probes have non-zero weights). Our approach builds on a recently proposed maximum margin clustering algorithm [21], [22], which brings ideas from large-margin supervised learning techniques like support vector machine classification and support vector regression to the unsupervised clustering problem; the choice of constraints was motivated by recent work on fused lasso regression [28] (see Methods).

Since each linear separator results in a binary partition of samples, we apply our procedure iteratively to separate each group of samples into two new groups in such a way that the new linear separator is orthogonal to the previously determined ones. Therefore, each step will find a new direction of variation in the aCGH data (similar to principal component analysis [29]), and the overall procedure results in a hierarchical partitioning of the data set (see Methods).

### Large-margin partitioning reveals hierarchy of copy number changes

We collected our data set from the Cancer Genome Atlas (TCGA) data portal [7]. It contains 345 glioblastoma tumor samples with copy number changes profiled on Agilent 244K arrays (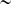
228K probes). This data set has previously been analyzed to determine major amplification and deletion events using the RAE [13] and GISTIC [12] algorithms [7].

We used the Level 2 data already produced by the previous analysis [7]. This data has already been normalized through the application of a lowess algorithm on the log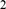 ratio data, and probes flagged as low-quality (saturated, non-uniform or faint) are excluded. Quality of the arrays was also measured through the proportion of excluded probes and the consistency of values associated with successive probes, and low-quality arrays were removed from the data set.

We ran our algorithm separately on every chromosome, with a sparseness coefficient 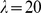 and a piecewise-constantness coefficient 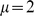 (see Methods). Empirically, we found the following dependence on the choice of these coefficients: if the coefficients were chosen to be too small, it would result in a trivial clustering, with all samples assigned to the same group; if the parameters were too permissive, the clustering obtained would be the same as standard 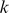-means (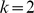). However, between these two extremes, clustering results were not overly sensitive to parameter choice. We expect the suitable range of parameters to depend on the array platform as well as statistical properties of the array profiles in a given data set. We therefore suggest performing a grid search on a subset of the samples and selecting the smallest possible parameters that give a non-trivial clustering on every chromosome.

In order to assess the significance of our results, we used a random model where we shuffled the probes of our dataset and compared the distance between the median samples of our two groups to the distribution of 1000 distances of median samples of two random sample groups separated with the same classifier. We verified that the randomized distance distribution follows a normal distribution, and we computed the 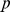-value for the distance between the median samples corresponding to the tail of this normal distribution.

For each chromosome, we constructed a “clustering tree” by iteratively splitting each group into two if it respected three criteria. The first criterion was that it must contain more than five samples (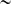
1.5% of the data set), since it would be difficult to achieve a statistically significant partition of very small subsets. The second criterion was that splitting this group would not make the depth of our tree bigger than 3. The maximal depth was chosen heuristically: after three iterations, we empirically found that the groups were too small or the separation was not significant anymore. The last criterion was that the partition generating this group must satisfy a significance threshold of 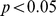. While this 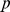-value may seem overly permissive, it is important to understand that our estimator (the centroid distance) is not directly optimized by the algorithm; therefore, the empirical 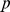-values generated are fairly conservative.

**Figure 2:**
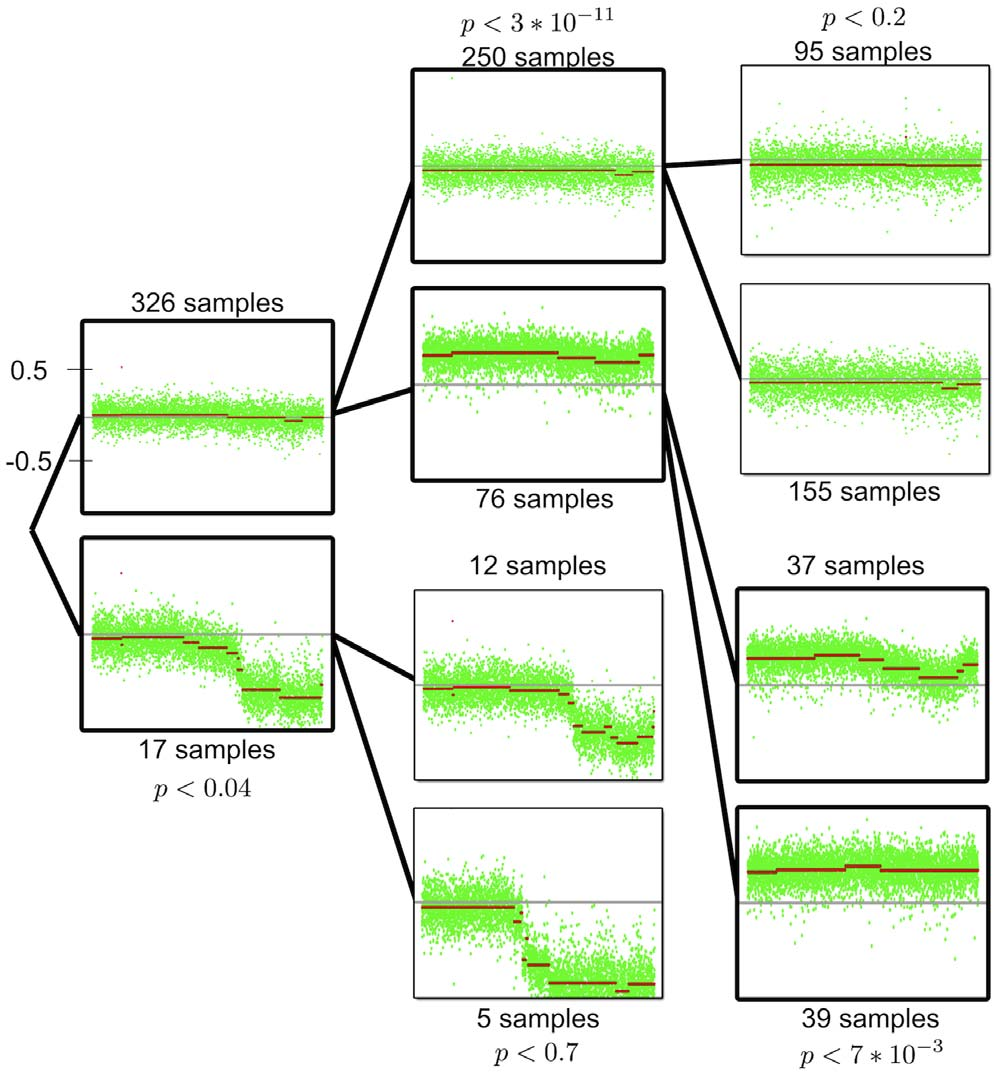
Clustering tree for chromosome 19. At each iteration of the algorithm, each previously identified group of samples are partitioned into two new clusters used a maximum-margin clustering technique that exploits the correlations in aCGH profiles (see Methods). The partitioning process stops when (i) a group has fewer than 5 samples; (ii) the partition generating the group fails to achieve a statistical significance threshold of 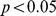; or (iii) the tree is already at the maximum depth of 3. In the picture above, each group is represented by its centroid, i.e. its median profile, in green. For visualization purposes, the segmentation of the centroid, produced by circular binary segmentation [30], is shown in red.

Figure 2 gives an example of a “clustering tree” produced by our algorithm for chromosome 19. The first iteration separates the samples into two clusters, one with 17 samples that presents a deletion of a region of the q arm and one of 326 samples, with 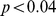. The centroid of each cluster is shown in green (Figure 2, leftmost column); in addition, a segmentation of each cluster centroid using a standard tool (circular binary segmentation [30]) is shown to aid visualization of the copy number differences between the two groups. As 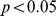 for this separation and each cluster is bigger than 5 samples, we split each of these subsets into two new groups. The splitting of the group of 17 samples is is not associated with a significant enough median separation (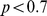) and therefore is not split again. On the other hand, the partition of the group of 326 samples produces one group of 250 samples without any apparent significant CNA and a group of 76 samples whose centroid shows an amplification of the whole chromosome. This split has strong significance (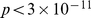), and therefore both of these groups are split again. The partition of the group of 250 samples does not achieve significance (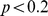), and neither of the resulting clusters show any significant CNAs. The group of 76 samples is divided into two new groups of 37 and 39 samples (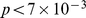). Each of these groups shows an amplification of the whole chromosome, but the group with 39 samples seems to have a lower amplification of the q arm than of the p arm while the other does not. As we limit ourselves to trees of depth 3, we do not partition either of these groups any further.

### Analysis of glioblastoma aCGH data recovers known CNAs without segmenting samples

We applied the iterative procedure to each chromosome independently, as described in the previous section. To call characteristic CNAs of each cluster, we applied circular binary segmentation [30] using default parameters on its centroid, i.e. the median profile of the cluster, and associated the characteristic CNA(s) of this centroid to the cluster. One should understand that the aberrations of the centroid profile may not be shared by every one of the cluster samples, but that it gives a good estimate of these events. We also caution that the size of the partition gives a good idea of the penetrance but is not entirely equivalent.

The first iteration of our algorithm found an amplification of the whole chromosome 1, of the whole chromosome 7 and of the whole chromosome 20. It also identified the deletion of the whole 9p arm, as well as a big part of 19q, the whole chromosome 10, the whole chromosome 13, the whole chromosome 14 and the whole chromosome 22. The second iteration of the algorithm found the loss of 6q arm, deletion of the whole chromosome 15, of the whole chromosome 16 and an amplification of the whole chromosome 19. It also demonstrated that some samples that present an amplification of chromosome 7 also contain a focal and very strong amplification event on the 7p arm. The third iteration of the algorithm identified focal amplification events on chromosome 3 and on chromosome 4. It also showed a loss of the whole chromosomes 9 and 21. These results are summarized in Table 1, along with the size of the partition in which each CNA was identified in terms of number of samples and percentage of the full data set.

**Table 1:** Summary of significant events in glioblastoma data set.

Event	Iter.	# of samples	% of samples	Size of event	Correlated genes	Examples of candidate genes
(a) Chr. 1	1	26	7.5%	247 Mbp	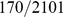	LCK, PAX7, RPL22
(a) 3q26.1	3	6	1.7%	25 Kbp	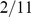	
(a) 4q12	3	7	2.0%	236 Kbp	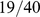	CHIC2, FIP1L1, KIT, PDGFRA
(d) 6q	2	31	9%	110 Mbp	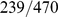	FOXO3A
(a) Chr. 7	1	169	49%	158 Mbp	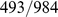	BRAF, CDK6, EGFR, ELN, HIP1, PMS2, SMO, TIF1
(a) 7p11.2	2	76	22%	37 Kbp	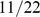	EGFR
(d) 9p	1	99	29%	47 Mbp	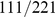	CDKN2A- p14ARF, CDKN2A -p16(INK4a),FANCG, JAK2, MLLT3, PSIP2
**(d) Chr. 9**	3	7	2%	140 Mbp	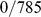	
(d) Chr. 10	1	154	45%	135Mbp	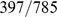	BMPR1A, D10S70, MYST4, NCOA4, PTEN, SSH3BP1
(d) Chr. 13	1	61	18%	114Mbp	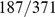	ERCC5, FOXO1A, LHFP, RB1, ZNF198
(d) Chr. 14	1	165	48%	106Mbp	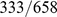	AKT1, BCL11B, DICER1, GPHN, KTN1, TCL1A, TCL6, TSHR
**(d) Chr. 15**	2	21	6.1%	100 Mbp	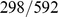	BLM, CRTC3, NTRK3, PML
**(d) Chr. 16**	2	15	4.3%	88.8 Mbp	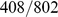	CBFB, CDH1, CREBBP, CYLD, HERPUD1, IL21R, CDH11, MAF, MHC2TA, MYH11, TNFRSF17
**(d) 19q13.2–19q13.43**	1	17	4.9%	25.2 Mbp	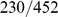	BCL3, ERCC2, TFPT, ZNF331
(a) Chr. 19	2	76	22%	63.8 Mbp	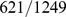	AKT2, BCL3, BRD4, CIC, ELL, ERCC2, KLK2, SH3GL1, STK11, TCF3, TFPT, TPM4, ZNF331
(a) Chr. 20	1	74	21%	62.4 Mbp	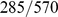	ASXL1, GNAS, SS18L1, TOP1
**(d) Chr. 21**	3	6	1.7%	46.9 Mbp	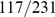	ERG, RUNX1, DSCR1
(d) Chr. 22	1	300	87%	49.7 Mbp	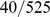	CTCL1, EWSR1, MKL1, SMRCB1, ZNF278

We indicated the iteration in which the event was found as well as the number of samples that were assigned to this cluster and the percentage of the total number of samples this represented. Deletions are denoted by the symbol (d) and amplifications by the symbol (a). Region names in boldface denote novel CNAs that were not found by previous analyses while underlined regions represent short events. Candidate genes denote significantly differentially overexpressed genes in this region if the CNA is an amplification and significantly differentially underepxressed genes in this region if the CNA is a deletion, according to a SAM analysis and out of the total number of genes in the region.

**Figure 3:**
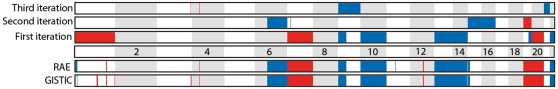
Comparison of the gains and losses found by iterative partitioning versus previous analyses. The horizontal tracks show the CNAs identified by first three iterations of our method, compared to the ones found by GISTIC and RAE. The middle track depicts the chromosomes, with even chromosome numbers annotated. Gains are denoted in red and losses in blue.

An analysis of the same data set using both RAE [13] and GISTIC [12] algorithms has already been published [7]. Both methods agreed on significant large-scale amplification events for the whole chromosomes 7, 19 and 20 and focal amplification events on chromosome 1 and 12; significant large-scale deletion events on chromosomal arms 6q, 9p, 15q, on whole chromosomes 10, 13, 14 and 22; and focal deletion events on chromosome 1. In addition, RAE found significant focal amplification events on chromosome 14, as well as significant focal deletion events on chromosome 11. By contrast, GISTIC found different additional focal amplification events on chromosomes 3 and 4. Figure 3 includes a summary of our results as well as a comparison with the amplification and deletion events found by both of these analysis.

As shown in Figure 3, most of the events found in both RAE and GISTIC analyses are found by the first two iterations of our method, including every large-scale event identified by these methods. Exceptions include a small amplification event on chromosome 12, the events on chromosome 1 (where our method disagrees with the finding of RAE and GISTIC) and an amplification event on chromosome 4, which is found on our third iteration.

### Iterative partitioning reveals novel CNAs supported by independent glioblastoma studies

Beyond recovering almost all the CNAs identified by methods like RAE and GISTIC, our iterative partitioning algorithm found a number of significant events that were not discovered by previous analyses of this dataset. These events include an amplification of the whole chromosome 1, a deletion event on the whole chromosomes 9, 15, 16 and 21, as well as a deletion of the 19q arm.

Some of these events have been documented in studies of independent copy number data sets, such as the deletion on the 19q arm [31], [32] and of chromosome 16 [33]. The deletion of chromosome 21 has been previously associated with glioblastoma [34], and it has been proposed that the low incidence of glioblastoma in Down's syndrome patients is linked to the chromosome 21 trisomy that characterizes this genetic condition [35]. Here, we find the chromosome deletion associated with a very small cluster (6 samples), and the low frequency presumably explains why this aberration was missed by previous analyses. The deletion of chromosome 15 actually includes the deletion on the 15q arm found in the previous analyses. The shape of the centroid for this partition shows that the amplitude of the deletion is smaller on the rest of the q arm and on the p arm, and it is possible that full chromosome deletion was not found by RAE or GISTIC due to the smaller amplitude.

To identify genes that are well correlated with the CNAs, we performed a significance analysis of microarray (SAM) using the SAMR package. For each cluster, we labeled each sample according to its label (inside or outside the cluster of interest) and looked at the number of genes of the region of the CNA that were significantly differentially underexpressed in the case of a deletion, or significantly overexpressed in the case of an amplification. Calculations were done using the t-statistic, 100 permutations and the Tusher method [36].

Our results, summarized in Table 1, show that in most cases a large number of genes had expression levels that are significantly correlated with the assignment of samples to the cluster harboring the CNA. It should be noted that the relationship between expression and copy number is complex, and that the absence of significant correlations does not exclude the presence of the CNA, especially in cases where the low count of genes or samples makes this correlation statistically difficult to prove.

The novel CNAs discovered by our analysis are correlated with several important genes. For example, the deletion of the chromosome 16, the 19q13.2–19q13.43 regions, and the chromosome 21 are significantly correlated with underexpression of candidate cancer-suppressor genes, respectively CBFB [37], [38] or CDH11 [39], TFPT [40] and DSCR1 [35], giving additional evidence in support of these events.

### Several sets of frequent chromosomal aberrations show high correlation

One advantage of our method compared to score-based approaches such as RAE and GISTIC is that it gives an assignment of samples to groups – or, more precisely, identifies CNAs by simultaneously finding the groups of samples that harbor them – which makes it easier to identify which samples are affected by which frequent CNAs. We associated each sample to a set of frequent CNAs based on its cluster assignments in the chromosome-based iterative partitioning procedure. We found that co-occurrences of frequent CNAs within a sample were common; indeed, a majority of samples (249 out of 345) contained 2 or more of the frequent CNAs listed in Table 1.

We further examined co-occurrences of pairs of frequent CNAs, and we found that 31 pairs can be considered correlated (i.e. with an intersection of sample assignment better than expected by background frequencies) with 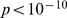 by Fisher's exact test (see Supplementary Figure S1).

A simple analysis of these significant pairs revealed that these correlated CNAs can actually be seen as three groups of co-occurences:

1. The amplification of chromosome 7 and its associated focal amplification event, the deletion on 9p, the deletion of chromosomes 10, 13 and 14 as well as the amplifications on chromosomes 19 and 20 are all highly correlated.
2. The deletion of 6q is well correlated with the focal amplification event on chromosome 7 as well as with the deletion on 9p.
3. The deletion on chromosome 22 is well correlated with the amplification of chromosome 7 (but not with the associated focal event), the deletion of chromosome 10 and the deletion of chromosome 14.

## Discussion

### Recovery of CNAs missed by summary statistics

Some of the novel glioblastoma CNAs that we found are good examples of how our method improves on summary statistic approaches, such as RAE and GISTIC. For instance, the deletion of chromosome 15 has only been spotted on the q arm by RAE and GISTIC. When we examined the profile of the centroid of a cluster identified by our method, we saw a lower amplitude deletion on the p arm as well. Because of this low amplitude, each probe on its own would not have a significant mean deletion across the data set and would hence be missed by a summary statistic. However, because all of the probes for the chromosome are affected, the deletion should be considered a significant CNA and is readily identified by approach.

As a second example, the deletion of the region 19q2–19q13.3 has not been found by other methods applied to the TCGA data set, even though it has been confirmed as a deletion event by previous studies. Here, the problem seems to be the fact that the same region is also present as an amplification event on a larger number of samples, which confounds the detection of this deletion by a summary test statistic. Finally, the deletion of the whole chromosome 21 is presumably missed by other methods because it is presents on only a small number of samples (6 samples or 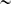
2%). However, since this event is a deletion of the whole chromosome and therefore supported on many probes, intuitively it should be much more statistically significant that a smaller but similarly infrequent event. Indeed, the importance of this CNA is confirmed by previous studies linking trisomy 21 in Down's syndrome to lower prevalence of glioblastoma as well as by the correlation with the under-expression of a candidate tumor-supressor gene present in this region.

### Recovery of focal events

Figure 3 shows that even though the first iteration of our algorithm seems to focus on large aberrations, the following iterations are able to find focal events such as the ones on chromosomes 3 and 4, and that our algorithm is therefore able to find focal events as well as large ones. The only focal event whose presence is agreed on by both RAE and GISTIC and that our method is not able to find is the one on chromosome 12. Looking at the raw data shows us that this event is shared by roughly 40 samples but only affects 2 probes, which makes it a difficult signal to find when looking a multiple probes. However, by restricting our analysis to a small interval centered on the event (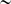
300kbp or 40 probes), we were able to identify the common event using our maximum-margin clustering algorithm (see Supplementary Figure S2), suggesting that our method could perhaps be used in conjunction with a sliding window to improve detection of very small events.

### Analysis of samples with high noise and genomic instability

The glioblastoma copy number profiles that we analyzed here have relatively few CNA events and therefore provide a favorable test case for computational analysis. Copy number data sets for other cancers have proven far more problematic. For example, a recent copy number study of lung adenocarcinoma [8] compiled a very large (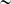
400 samples) but challenging data set, where the signal to noise varied considerably over samples – potentially due to stromal contamination – and a sizable fraction of samples displayed numerous events. The authors curated the samples into three tiers based on signal quality and restricted analysis to the best tier. Despite the large average number of events per samples, the study identified only a few regions altered in a significant number of samples, with the most common CNA (amplification of chromosome 14q13.3) only present in 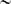
12% of the best third (top tier) of their samples. We applied our method to this lung adenocarcinoma data set to see how it would perform in a high noise setting. Since the original assignment of samples to tiers was not readily available, we did a first pass analysis of the entire data set – without attempting to reduce to the cleanest samples – using the same parameters as we used on the TCGA data set. Interestingly, the first iteration of the algorithm partitioned each chromosome into two clusters containing exactly the same samples (with 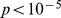), with one group consisting of samples with a strong but very noisy signal and the other containing samples with a weak signal. This result suggests that our method may be able to automatically distinguish signal quality.

The initial choice of parameters did not find any significant aberrations at a 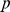-value cutoff of 0.05, possibly due to the different array platform as well as the different statistical properties of the copy number profiles (see Supplementary Figure S3 and Supplementary Table S1). However, using our algorithm with a different set of parameters (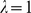 and 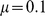) on chromosome 14 allowed us find the amplification of 14q13.3, albeit only in 6 samples (2% of the total count of samples) and with a weak 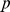-value (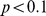). Here, the presence of a large group of very noisy samples in the data set may be responsible for degrading the 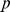-value. While we were not able to directly compare to the original analysis on the top tier samples, this quick analysis on the full data set is fairly encouraging, in that we were able to retrieve the main result without an *ad hoc* curation of samples.

### Possible algorithmic extensions

The above analysis also underscores the impact of the choice of the two constraint parameters, 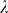 and 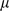 (see Methods), which determine the degree of sparseness and piecewise-constantness, respectively, of our linear classifiers. We chose the parameters for the glioblastoma study through heuristics and recovered most known events as well as several novel and plausible CNAs. However, full exploration of this parameter space could yield additional results; for example, to predispose the algorithm to find focal events, one might try to make the sparsity constraint more stringent. Various strategies might be used to optimize the choice of parameters, including use of a cross-validation loop. To implement this approach, one would have to choose an appropriate method for estimating the quality of the clusters: standard estimators are closely tied to the objective functions optimized by traditional clustering algorithms (such as 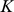-means), which do not take into account the properties of copy number profiles (i.e. spatial correlations, sparsity of deletion/amplication events). However, such a cross-validation loop would also entail lengthier computational times. This cost could be greatly reduced if we were able to compute the entire regularization path of the fused lasso in a single pass, as others were able to do with the original lasso [41] and SVM [42] optimization problems.

An interesting direction for future research would be to extend this method to incorporate gene expression data in the analysis of copy number profiles. The candidate gene results of Table 1 show that even a simple analysis is able to find significant correlations between the two types of data. Presumably, CNAs that result in deregulated expression are more likely to be driver mutations. A framework that integrates paired copy number and mRNA expression may yield greater insight into functional gains and losses in cancer.

### Conclusions

We have introduced a new mathematically sound method for the identification of frequent alterations in a large cohort of tumor copy number profiles. This method builds on the concept of maximum-margin clustering by extending to more than two groups and incorporating specific properties of copy number data, i.e. the piecewise-constantness and the sparsity of CNAs.

We applied this method to a large publicly available glioblastama data set from The Cancer Genome Atlas initiative. Our results include most CNAs already found by previous studies as well as novel CNAs confirmed by other data sets or expression analyses. We showed that we were able to identify large aberrations as well as focal events and found significant correlations between these different CNAs.

## Methods

Below, we briefly develop the technical background related to our approach and describe the details of our algorithm. We first present the fused lasso classification algorithm and then show how to extend it to an unsupervised setting based on the maximum margin clustering algorithms. Finally, we introduce our iterative partitioning procedure for determining hierarchical clusters characterized by common CNAs.

### Supervised classification

We first consider the supervised learning problems for aCGH profiles. Here we are given a training set of aCGH samples 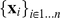 of dimension 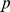, where 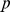 is the number of probes; each example 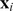 has an associated label or an explanatory variable 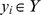, where the labels can be discrete (classification) or real-valued (regression).

Given our labeled set of samples, the goal of linear supervised classification or regression is to build a linear function 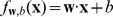 that will be able to predict the correct explanatory variable 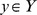 for a new sample 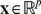. We use a general formulation of supervised learning as an optimization problem:
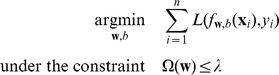
(1)where 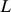 is a loss function that penalizes the error between the predictions 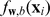 and the real explanatory variables 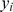, 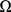 is a regularization function, and 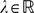 the value of the constraint, to be adjusted to find a suitable compromise between minimizing of the error term and regularizing (avoiding overfitting) the model.

Problem (1) describes a whole family of algorithms that includes (i) the support vector machine (SVM), when 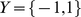 is the set of binary labels, 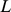 is the hinge loss 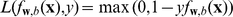, and 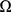 is the Euclidean norm; (ii) the L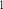-SVM, when 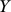 is the set of binary labels, 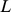 is the hinge loss, and 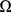 the L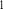-norm; or (iii) lasso regression, when 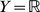, 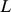 is the squared error 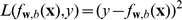, and 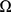 is the L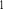-norm; among many others.

### Maximum margin clustering

Recently Xu et al. proposed to generalize this optimization framework to the unsupervised clustering problem, i.e. trying to find the best linear separator between (latent) classes of samples when the labels are not known [21]. The general optimization problem described in (1) then becomes
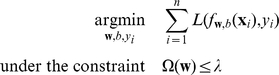
(2)


However, in the case of binary classification, i.e. 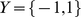, Problem (2) becomes a mixed integer problem (MIP), which is not easily solvable using standard optimization techniques. Instead, Zhang et al. proposed an algorithm similar to conjugate descent to solve this problem [22], alternating between (a) training the linear separator given current label assignments and (b) updating the label assignment based on the linear separator. They found that a standard support vector machine (SVM) converges quickly in this alternating procedure to a fixed set of labels without finding more favorable cluster assignments. Therefore, they proposed using support vector regression (SVR) for the linear separator. SVR is more often used in the case of regression, i.e. 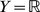, than in binary classification but performs well for the clustering problem.

### Incorporating prior knowledge

In choosing the regularization function 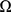 to use in training a linear separator, we want to take into account two different properties of copy number profiles:

1. Successive probes on the same chromosomes are likely to represent the same copy number and should therefore tend to be attributed similar weights in the linear function.
2. There are usually only a small number of CNAs in a given sample, often (but not always) occupying relatively small genomic regions, and therefore only a small number of probes should have non-zero weights in the linear function.

Tibshirani and Saunders introduced a fused lasso method for regression and classification that gives a sparse and piecewise-constant linear function by imposing two separate constraints [28]; the regression formulation takes the form:
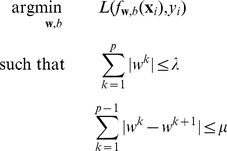
(3)where 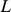 is the least squares loss function. Here, the first constraint is the lasso regularizer, which induces sparsity, i.e. few components 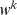 in the solution vector 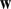 are non-zero; the second constraint enforces piecewise constantness, i.e. adjacent probes tend to be assigned the same weight.

In the case of high-density copy number profiles, another issue is the non-uniform distribution of the distances between successive probes [43]. Older low resolution aCGH technologies used probe sets designed to have relatively uniform inter-probe distances, or at least, these distances varied within an order of magnitude. New higher resolution technologies have higher disparities in inter-probe distances. To take these into account, we modify the constraints to include a coefficient that normalizes for inter-probe distances:
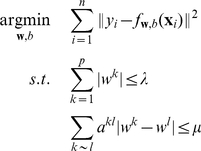
(4)where 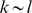 if 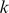 and 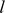 refer to succesive positions on the same chromosomal arm and 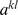 is the weight of the corresponding relation.

In the case of aCGH profiles, we define 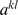 as
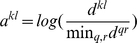
(5)where 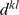 is the genomic distance between probes 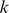 and 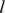.

Incorporating these modifications, we obtain the following quadratic problem under linear constraints:
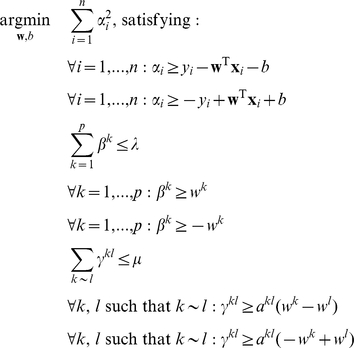
(6)


Using this quadratic problem, we propose an algorithm similar to the maximum margin clustering algorithm [22]:

#### Algorithm 1

*Iterative fused lasso*.

1. Initialize the labels 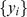, for example with standard 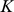-means (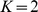).
2. Calculate the linear separator 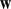 obtained by solving Problem (4).
3. Assign the labels using the linear separator: 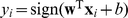.
4. Repeat steps 2–3 until convergence.

### Iterative partitioning

One limitation of the method proposed in Problem (4) is that it only achieves a binary partition of the data, while in fact there may be more than two distinct subgroups defined by common CNAs. In order to overcome this limitation, we use the following iterative partitioning algorithm:

#### Algorithm 2

*Iterative partitioning*.

1. Initialize the partition of the data with Algorithm 1.
2. Partition each of the groups of the partition into two new groups.
3. Repeat steps 2 until the size of a new group or the significance of the partition falls below threshold.

In order to guarantee that the newly discovered groups at each step will explore different directions of variation, we make each classifier orthogonal to the preceding ones. This can be done by the following equation, assuming that we know the classifiers 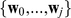, we can then learn a new classifier (and associated partitioning) 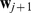, written as 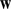 to simplify notation:
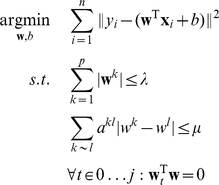
(7)where 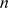 is the number of samples that we want to separate.

Using the same method as in the last section, Problem (7) can be transformed into a quadratic problem under linear constraints.

### Implementation

The method has been implemented under Matlab using the commercial Tomlab/CPLEX [44] package. Both this implementation and another one using the free SeDuMi [45] package are freely available.

## Supporting Information

Figure S1 Correlation matrix of frequent CNAs. The heatmap shows the significance of the correlation between pairs of CNAs in the TCGA glioblastoma data by displaying the p-value (Fisher's exact test) on a logarithmic scale. Every pair with p<1e-10 is given the same color. The size of each square is proportional to the size of the corresponding CNA (on a logarithmic scale).
(2.08 MB TIF)

Figure S2 Analysis around the short event on chromosome 11 in the TCGA glioblastoma data set. We performed our clustering algorithm on a small region of ∼300kbp (or 38 probes) centered around the small deletion event found by RAE and GISTIC on chromosome 11. The heatmap shows the value of the probes of the samples on this region, with green indicating negative values and red indicating positive values. The vertical axis represents the sequence of probes along the genome, while the different samples are shown on the horizontal axis. The blue and yellow color bars correspond to the labels of each sample as determined by the first iteration of our algorithm. These labels are perfectly correlated with the presence of the bright green deletion event.
(1.85 MB TIF)

Figure S3 Centroids of chromosome 14 clusters on the lung adenocarcinoma dataset. The figure shows the two centroids of the clusters found with the first iteration of our method on chromosome 14 in the lung adenocarcinoma data set. The larger probe signal amplitude and variance of the blue centroid (corresponding to the smaller group) show that this cluster's samples have stronger signal than the other cluster (see also Supplementary Table S1).
(0.71 MB TIF)

Table S1 Variance of the lung aCGH profiles. We present the mean probe signal variance of the samples of each cluster found at the first iteration on the lung adenocarcinoma data set, compared to the corresponding mean variance of clusters for the TCGA data set. As described in the main text, the lung clusters for different chromosomes always contain the same samples. The table shows that the cluster variance is also surprisingly regular, and that the the smaller group variance is especially big.
(0.04 MB PDF)
