# Supplementary figures and images for: Determining Frequent Patterns of Copy Number Alterations in Cancer
Source: PLoS One. 2010 Aug 12;5(8):e12028. doi: 10.1371/journal.pone.0012028 (PMC2920822; doi:10.1371/journal.pone.0012028)

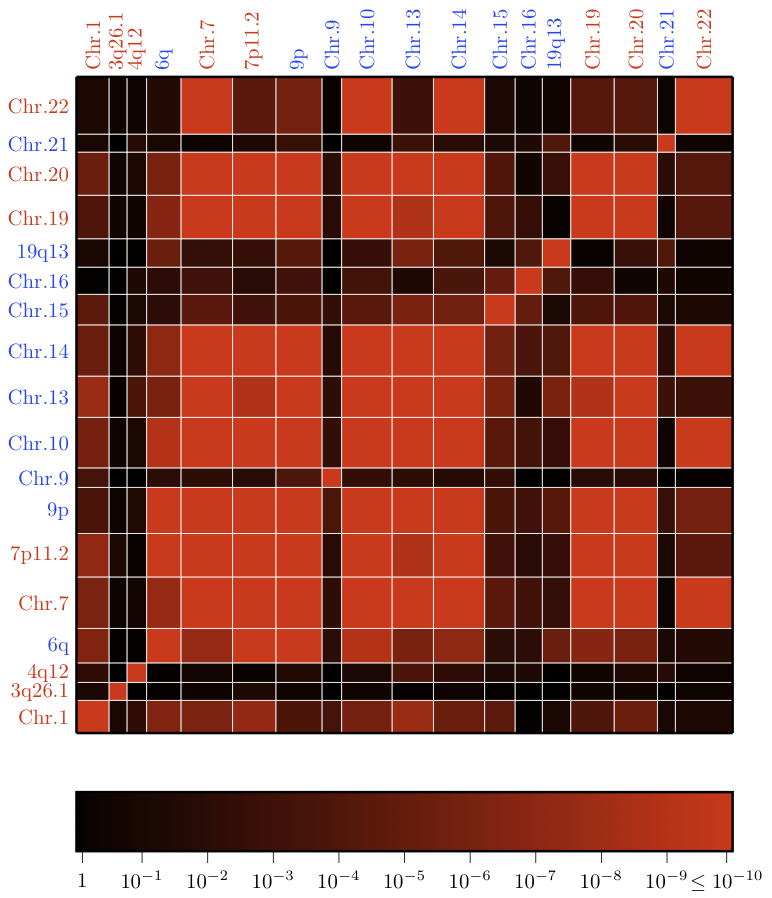

Supplement: Figure S1 — Correlation matrix of frequent CNAs. The heatmap shows the significance of the correlation between pairs of CNAs in the TCGA glioblastoma data by displaying the p-value (Fisher's exact test) on a logarithmic scale. Every pair with p<1e-10 is given the same color. The size of each square is proportional to the size of the corresponding CNA (on a logarithmic scale). (2.08 MB TIF) [file pone.0012028.s001.tif]

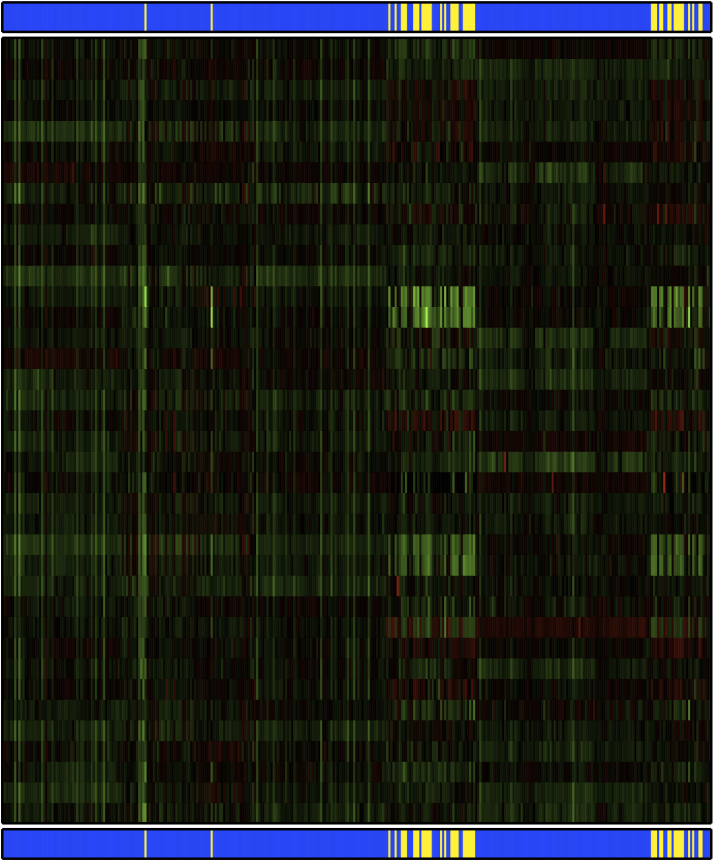

Supplement: Figure S2 — Analysis around the short event on chromosome 11 in the TCGA glioblastoma data set. We performed our clustering algorithm on a small region of ∼300kbp (or 38 probes) centered around the small deletion event found by RAE and GISTIC on chromosome 11. The heatmap shows the value of the probes of the samples on this region, with green indicating negative values and red indicating positive values. The vertical axis represents the sequence of probes along the genome, while the different samples are shown on the horizontal axis. The blue and yellow color bars correspond to the labels of each sample as determined by the first iteration of our algorithm. These labels are perfectly correlated with the presence of the bright green deletion event. (1.85 MB TIF) [file pone.0012028.s002.tif]

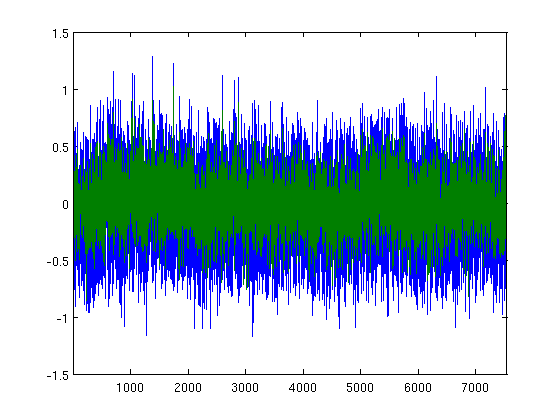

Supplement: Figure S3 — Centroids of chromosome 14 clusters on the lung adenocarcinoma dataset. The figure shows the two centroids of the clusters found with the first iteration of our method on chromosome 14 in the lung adenocarcinoma data set. The larger probe signal amplitude and variance of the blue centroid (corresponding to the smaller group) show that this cluster's samples have stronger signal than the other cluster (see also Supplementary Table S1). (0.71 MB TIF) [file pone.0012028.s003.tif]
