# Supplementary material for: Determining Frequent Patterns of Copy Number Alterations in Cancer
Source: PLoS One. 2010 Aug 12;5(8):e12028. doi: 10.1371/journal.pone.0012028 (PMC2920822; doi:10.1371/journal.pone.0012028)
Supplement: Table S1 — Variance of the lung aCGH profiles. We present the mean probe signal variance of the samples of each cluster found at the first iteration on the lung adenocarcinoma data set, compared to the corresponding mean variance of clusters for the TCGA data set. As described in the main text, the lung clusters for different chromosomes always contain the same samples. The table shows that the cluster variance is also surprisingly regular, and that the the smaller group variance is especially big. (0.04 MB PDF) [file pone.0012028.s004.pdf]

Chr.	var. big group (lung)	var. small group (lung)	var. big group (TCGA)	size big group (TCGA)	var. small group (TCGA)	size small group (TCGA)
1	0.0898	0.2073	0.0679	317	0.101	26
2	0.0829	0.2044	0.052	188	0.0514	155
3	0.0871	0.2031	0.059	331	0.0629	12
4	0.0823	1.999	0.0713	188	0.0543	155
5	0.0939	0.2113	0.0547	187	0.0513	156
6	0.0861	0.2055	0.059	177	0.0774	166
7	0.0916	0.2138	0.0667	174	0.1228	169
8	0.0957	0.2104	0.0598	300	0.0867	43
9	0.0814	0.1972	0.0634	244	0.2348	99
10	0.0805	0.2009	0.0636	189	0.0685	154
11	0.083	0.2029	0.0571	300	0.0998	43
12	0.0866	0.2059	0.0817	331	0.0653	12
13	0.0811	0.1967	0.0557	282	0.123	61
14	0.0865	0.203	0.0591	178	0.0714	165
15	0.0798	0.2048	0.0588	187	0.0569	156
16	0.0839	0.2034	0.0595	193	0.0568	150
17	0.0863	0.2087	0.0615	190	0.0535	153
18	0.0822	0.1959	0.0451	330	0.1283	13
19	0.0899	0.2036	0.0682	326	0.1916	17
20	0.0868	0.2108	0.0552	269	0.0557	74
21	0.0795	0.2022	0.0523	187	0.0547	156
22	0.0796	0.2042	0.0635	300	0.127	43
**mean**	0.0852	0.286	0.0606	244	0.0906	99
